# Association between sleep disorder and quality of life in patients with type 2 diabetes: a cross-sectional study

**DOI:** 10.1186/s12902-020-00579-4

**Published:** 2020-06-30

**Authors:** Yoshitaka Hashimoto, Ryosuke Sakai, Kenichiro Ikeda, Michiaki Fukui

**Affiliations:** 1 Department of Endocrinology and Metabolism, Kyoto Prefectural University of Medicine, Graduate School of Medical Science 465 Kajii-cho, Kawaramachi-Hirokoji, Kamigyo-ku, Kyoto, 602-8566 Japan; 2 Product Branding, Product Marketing Department, Kowa Company, Ltd., Tokyo, Japan

**Keywords:** Type 2 diabetes, Quality of life, Sleep disorder, Sleep quality, Sleep quantity, Sleep fragmentation

## Abstract

**Background:**

We investigated the association between sleep symptoms, which cause sleep disorder, and quality of life (QoL) among people with type 2 diabetes (T2D).

**Methods:**

In this cross-sectional study of 342 people with T2D, the Japan National Health and Wellness Survey (NHWS) database 2016 were used. We treated the respondents who reported experiencing any of the sleep symptoms as having sleep disorders. To examine health-related QoL (HRQoL), we used the physical component summary (PCS) and the mental component summary (MCS) from the 36-Item short-form and the EuroQol 5 Dimension (EQ-5D) survey instruments. Overall activity impairment was used for assessment of the effect on the individual’s ability to perform regular daily activities. We used t-test and one-way ANOVA test for comparison QoL scores between the participants with and without sleep disorders.

**Results:**

66.4% of the participants with T2D reported having a sleep disorder. The PCS, MCS, EQ-5D, and overall activity impairment of people with sleep disorder was significantly poorer than those of the people without. Specific sleep symptoms, such as waking up to go to the bathroom, daytime sleepiness, and waking up too early (before the alarm clock), had high prevalence (35.4, 27.8 and 20.2%). The participants who experienced waking up to go to the bathroom or daytime sleepiness demonstrated significantly poorer QoL on all scores related to QoL, but those who experienced waking up too early only demonstrated significantly poorer QoL on the EQ-5D.

**Conclusions:**

Two-thirds of people with T2D in this study suffer from sleep disorders. The people who experience waking up to go to the bathroom or daytime sleepiness had significantly poorer QoL than those without these symptoms. Thus, sleep disorders, especially the symptoms of waking up to go to the bathroom or daytime sleepiness, might be the treatment targets for QOL of people with T2DM.

## Background

The number of people with type 2 diabetes (T2D) is now increasing. The overall treatment goal of diabetes is to prevent diabetic complications, while maintaining quality of life (QoL). Reports show that the QoL of people with diabetes is lower than that of people without diabetes [1]. Health-related QoL (HRQoL) includes aspects of QoL that may affect physical or mental health [2]. Furthermore, there is a close association between HRQoL values and glycemic control in people with T2D [3, 4]. Thus, to prevent a decline in the QoL of people with T2D is treatment target for the management of diabetes.

Sleep disorder, which many people with T2D suffer, [5] is one of the factors impairing QoL in people with T2D. According to an internet survey of over 7 thousands people in the United States with T2D, three-quarters of participants suffered from sleep symptoms, and one-quarter of them were actually diagnosed with a sleep disorder [6]. Also, reports show that about half of people with T2D in Japan have sleep disorder [7]. Furthermore, in a study of almost 1 thousand Chinese people with T2D, 33.6% suffered from poor sleep quality, and there was an association between the pittsburgh sleep quality index and QoL [8]. These studies were based on the sleep disorder scores. These scores and sleep duration significantly correlate with QoL values in people with T2D [9, 10]. Generally, these sleep disorder scores were meaning the summary of symptoms in previous studies. We couldn’t specify which symptoms impact on patient’s QoL. Thus, we investigated the relationship between specific sleep-related symptoms and QoL in people with T2D.

## Methods

### Study design and participants

**Fig. 1:**
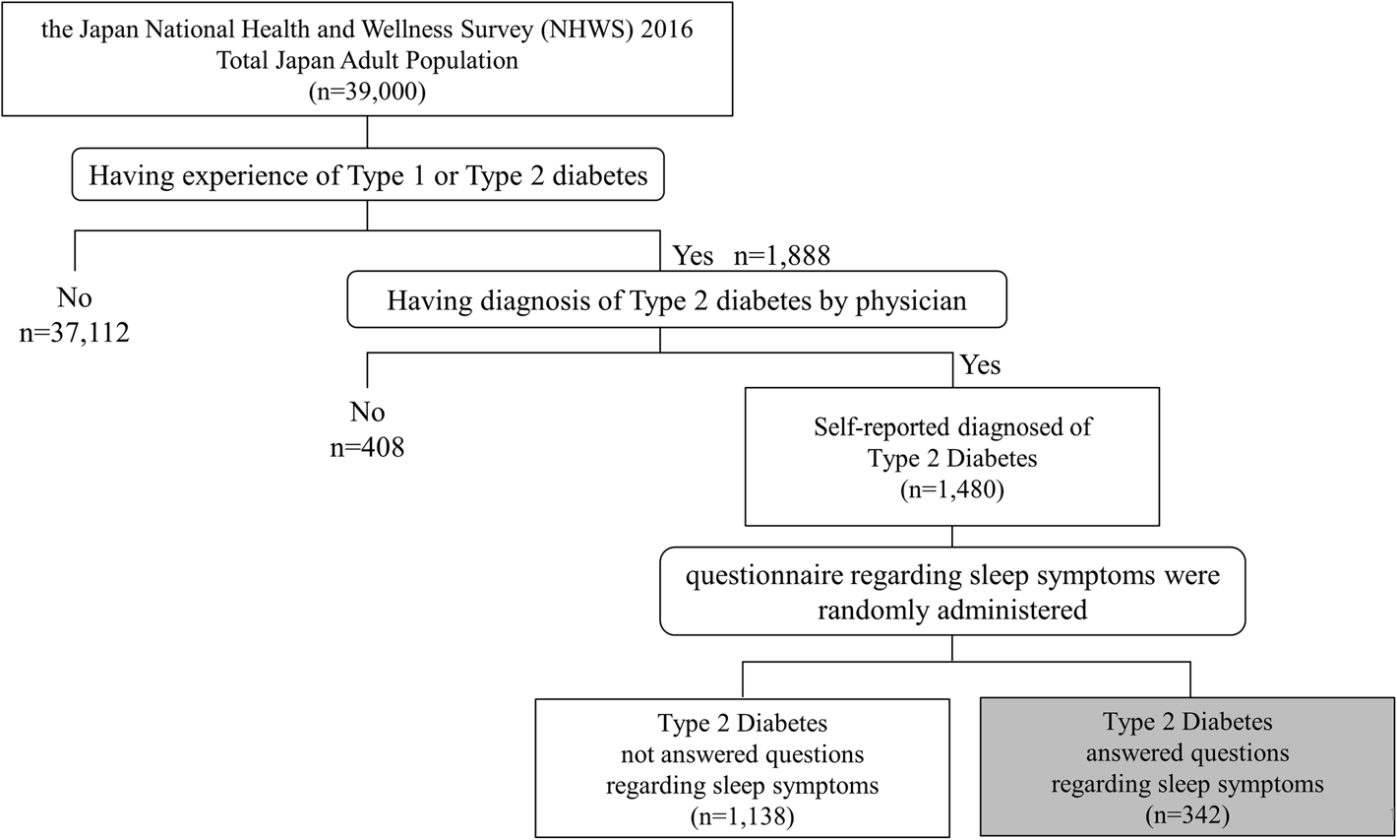
Data extraction from the Japan NHWS 2016

This cross-sectional study is based on the data of the Japan National Health and Wellness Survey (NHWS) 2016 (*N* = 39,000), which is a self-administered, internet-based questionnaire from a nationwide sample of adults (aged 18 years or older). In this survey, participants, were recruited through a random sampling approach, stratified by age and gender, are required to provide various information [11, 12]. The survey received Institutional Review Board approval and all respondents provided written informed consent prior to participating. Potential respondents for this study were identified through the general panel of Lightspeed Research (LSR). Panel members are general people who explicitly agreed to join this panel. The LSR members were recruited through a variety of means, including co-registration with other internet panels, e-newsletter campaigns, and banner placements. The NHWS sample is generally comparable to the greater population concerning these characteristics [11–13]. We extracted the data including self-reported diagnosis of type 2 diabetes from the Japan NHWS 2016. At first, all respondents were asked if they have ever experienced Type 1 or Type 2 Diabetes. For respondents who responded “yes” to this question, they were asked if their Type 2 Diabetes has been diagnosed by a physician. Respondents who answered “yes” to the second question were classified as T2D patients in the study. Among this T2D patients, we selected the individuals who answered questions regarding sleep symptoms. In addition, the questions about sleep symptoms were randomly administrated from all participants in the Japan NHWS 2016 (Fig. 1).

### Clinical and sociodemographic profiles

Using the questionnaire, age, sex, marital status (married/living with partner, or not married), education (university degree or all others), household income (< 3,000,000 JPY, 3,000,000 to < 5,000,000 JPY, 5,000,000 to < 8,000,000 JPY, 8,000,000 JPY or more, or decline to answer), health insurance (national health insurance, social insurance, late stage elderly insurance, or other/no insurance), and employment status (currently employed or not). Additional questions include: smoking (current, former, or never smoker), exercise (currently exercise or not), alcohol use (currently consume alcohol or not), body mass index (BMI) category (underweight; BMI < 18.5 kg/m^2^, normal weight; 18.5 kg/m^2^ ≤ BMI < 25 kg/m^2^, overweight; 25 kg/m^2^ ≤ BMI < 30 kg/m^2^, obese; 30 kg/m^2^ ≤ BMI, or decline to answer), taking steps to lose weight (yes or no), Charlson comorbidity index (CCI), HbA1c level (HbA1c < 7, HbA1c ≥7, or don’t know), use of injectable medicines (yes or no), and ever experienced hypoglycemia (yes, no, or don’t know). BMI (kg/m^2^) was calculated as person’s weight (kg) divided by the square of height (m^2^).

### Definition of sleep disorders

This study treats respondents who reported experiencing any of the sleep symptoms as having sleep disorders [6]. Sleep symptoms were made referencing the Diagnostic and Statistical Manual of Mental Disorders [14]”**.**

### Definition of QoL

HRQoL was assessed using the 36-Item short-form (SF-36v2) and the EuroQol 5 Dimension (EQ-5D) survey instruments. The SF-36v2 is a multipurpose, generic HRQoL instrument comprising 36 questions that map onto 8 health domains, and these relevant scores are summarized into the physical component summary (PCS) and the mental component summary (MCS) [15–17]. Higher scores indicate a better QoL. Validation of these score was confirmed in Japanese population [16]. The EQ-5D is an instrument that evaluates the generic QoL, which is a standardized measure of health status to provide a simple, generic measure of health [18]. EQ-5D were widely used in various countries and diseases [19–23]. This questionnaire and descriptive system comprising five dimensions (mobility, self-care, usual activities, pain/discomfort, and anxiety/depression) with five levels of severity [24] and then responses were converted into a single health index by applying a formula [24]. Higher scores indicate a better health status. Overall activity impairment was assessed using the Work Productivity and Activity Impairment (WPAI) questionnaire [25]. The WPAI was validated in number of disease states [25, 26] This is a 6-item validated instrument that consists of four metrics: presenteeism, absenteeism, overall work productivity loss, and overall activity impairment. Overall activity impairment is one of the WPAI’s metrics and is showing the percentage of impairment in daily activities because of one’s health in the past 7 days. Overall activity impairment subscales are expressed as percentages and higher percentages indicate greater impairment.

### Statistical analysis

We compared demographic and general health characteristics with and without sleep disorders. We also compared QoL, including HRQoL and overall activity impairment, based on the presence or absence of sleep disorders and of specific sleep symptoms. T-test and one-way ANOVA test were used for continuous variables and the Chi-square test for categorical variables.

Subsequently, the QoL associated with the presence or absence of sleep disorders or of specific sleep symptoms were compared by multivariable analysis (generalized linear model), adjusting for age, sex, marital status, level of education, Charlson comorbidity index, smoking status, alcohol use, BMI category, taking steps to lose weight, duration of diabetes, HbA1c level, use of injectable medications, and experience with hypoglycemia. We selected the specific sleep symptoms of waking up to go to the bathroom, daytime sleepiness, and waking up too early (such as before the alarm clock) for multivariable analysis because the prevalence rates of these symptoms were over 20%.

There was a significant difference when the *p*-value was less than 0.05. All data analyses were performed using IBM SPSS Statistics Version 22.

## Results

According to the Japan NHWS database 2016 (*N* = 39,000), 1480 participants were identified as having T2D. Among these T2D, the questionnaire about condition of sleep were randomly applied. Three hundred-forty two participants responded the questionnaire, and we analyzed the data of these respondents.

Table 1 shows the clinical characteristics of the 342 participants. The mean (SD) age and duration of diabetes were 63.25 (10.21) and 13.54 (10.59) years, and 82.5% of the participants were men. Out of the 342 participants, 15.5% used injectables and 27.8% had experienced hypoglycemia.

**Table 1:** Clinical and sociodemographic profile of study participants

	*N* = 342
Age (years)	63.25 (10.21)
Male	82.5% (282)
Marital status	
Married/living with partner	74.9% (256)
Divorced/separated/widowed	25.1% (86)
Education	
University degree	46.5% (159)
All others	53.5% (183)
Household income	
< 3,000,000JPY	17.0% (58)
3,000,000 to < 5,000,000 JPY	28.1% (96)
5,000,000 to < 8,000,000 JPY	24.0% (82)
8,000,000 JPY or more	18.7% (64)
Decline to answer	12.3% (42)
Insurance	
National health insurance	57.0% (195)
Social insurance	30.7% (105)
Late stage elderly insurance	8.8% (30)
Other/No insurance	3.5% (12)
Worker	46.2% (158)
Charlson comorbidity index	0.45 (0.91)
Smoking status	
Current smoker	26.3% (90)
Former smoker	38.9% (133)
Never smoker	34.8% (119)
Currently exercisers	55.0% (188)
Alcohol drinker	69.3% (237)
BMI category	
Underweight (< 18.5)	4.1% (14)
Normal weight (18.5 to < 25)	55.3% (189)
Overweight (25 to < 30)	27.2% (93)
Obese (≥30)	11.1% (38)
Decline to answer	2.3% (8)
Taking steps to lose weight	35.4% (121)
Duration of type 2 diabetes (years)	13.54 (10.59)
HbA1c level	
HbA1c < 7	37.1% (127)
HbA1c ≥7	41.8% (143)
Don’t know/Decline to answer	21.1% (72)
Use of injectable medications	15.5% (53)
Ever experienced hypoglycemia	
Experienced hypoglycemia	27.8% (95)
Not experienced hypoglycemia	63.5% (217)
Do not know	8.8% (30)

Continuous variables are expressed as mean (SD) and categorical variables are expressed as % (number)

Table 2 shows the prevalence of sleep symptoms in the participants. 66.4% reported some sleep symptoms. For individual symptoms, 35.4% wake up to go to the bathroom, 27.8% suffer daytime sleepiness, and 20.2% wake up too early (such as before the alarm clock).

**Table 2:** The prevalence of sleep symptoms

	*N* = 342
Experience any sleep symptoms	66.4% (227)
Difficulty falling asleep	12.6% (43)
Waking during the night and not being able to get back to sleep	12.3% (42)
Waking up several times during the night	13.2% (45)
Waking up too early (such as before the alarm clock)	20.2% (69)
Sleep apnea (temporary absence of breathing)	9.1% (31)
Leg cramps/leg problems	9.9% (34)
Waking up to go to the bathroom	35.4% (121)
Night sweats/hot flashes	5.8% (20)
Pain	0.9% (3)
Poor quality of sleep	12.0% (41)
Daytime sleepiness	27.8% (95)
Difficulty staying awake	2.3% (8)
Other	4.1% (14)

The data were expressed as % (number)

Table 3 shows the clinical characteristics difference between participants with/without sleep disorders. The proportion of current smoker, worse HbA1c, and experience with hypoglycemia were higher in the participants with sleep disorders than those in the participants without.

**Table 3:** Clinical and sociodemographic profile according to the experience of any sleep symptoms

	Any sleep symptoms (−) (*n* = 115)	Any sleep symptoms (+) (*n* = 227)	*p*-value
Age (years)	63.6 (9.61)	63.08 (10.52)	0.66
Male	81.7% (94)	82.8% (188)	0.804
Marital status			0.068
Married/living with partner	80.9 (93)	71.8% (163)	
Divorced/separated/widowed	19.1% (22)	28.2% (64)	
Education			0.306
University degree	42.6% (49)	48.5% (110)	
All others	57.4% (66)	51.5% (117)	
Household income			0.018
< 3,000,000JPY	18.3% (21)	16.3% (37)	
3,000,000 to < 5,000,000 JPY	21.7% (25)	31.3% (71)	
5,000,000 to < 8,000,000 JPY	24.3% (28)	23.8% (54)	
8,000,000 JPY or more	15.7% (18)	20.3% (46)	
Decline to answer	20.0% (23)	8.4% (19)	
Insurance			0.813
National health insurance	59.1% (68)	55.9% (127)	
Social insurance	27.8% (32)	32.2% (73)	
Late stage elderly insurance	8.7% (10)	8.8% (20)	
Other/No insurance	4.3% (5)	3.1% (7)	
Worker	40.0% (46)	49.3% (112)	0.102
Charlson comorbidity index	0.27 (0.61)	0.55 (1.01)	0.008
Smoking status			0.007
Current smoker	22.6% (26)	28.2% (64)	
Former smoker	31.3% (36)	42.7% (97)	
Never smoker	46.1% (53)	29.1% (66)	
Currently exerciser	55.7% (64)	54.6% (124)	0.857
Alcohol drinker	65.2% (75)	71.4% (162)	0.244
BMI category			0.131
Underweight (< 18.5)	6.1% (7)	3.1% (7)	
Normal weight (18.5 to < 25)	60.9% (70)	52.4% (119)	
Overweight (25 to < 30)	20.9% (24)	30.4% (69)	
Obese (≥30)	8.7% (10)	12.3% (28)	
Decline to answer	3.5% (4)	1.8% (4)	
Taking steps to lose weight	28.7% (33)	38.8% (88)	0.066
Duration of T2DM (years)	13.83 (10.63)	13.39 (10.59)	0.721
HbA1c level			0.008
HbA1c < 7	34.8% (40)	38.3% (87)	
HbA1c ≥7	34.8% (40)	45.4% (103)	
Don’t know/Decline to answer	30.4% (35)	16.3% (37)	
Use of injectable medications	11.3% (13)	17.6% (40)	0.127
Ever experienced hypoglycemia			0.001
Experienced hypoglycemia	14.8% (17)	34.4% (78)	
Not experienced hypoglycemia	74.8% (86)	57.7% (131)	
Do not know	10.4% (12)	7.9% (18)	

Continues variables were expressed as mean (SD) and categorical variables were expressed as % (number). T-test was used for continuous variables, and Chi-square test was used for categorical variables

Table 4 shows the QoL difference between participants with/without sleep disorders. The PCS, MCS, EQ-5D of the participants with sleep disorders were lower than those of the participants without. In addition, overall activity impairment of participants with sleep disorders was significantly higher than that of participants without.

**Table 4:** QoL scores according to the experience of any sleep symptoms

	All (***n*** = 342)	Any sleep symptoms (−) (***n*** = 115)	Any sleep symptoms (+) (***n*** = 227)	***p***-value
QoL score				
PCS	48.71 (7.40)	50.59 (6.71)	47.76 (7.56)	< 0.001
MCS	48.15 (10.59)	51.90 (8.36)	46.25 (11.10)	< 0.001
EQ-5D	0.79 (0.17)	0.86 (0.15)	0.76 (0.18)	< 0.001
Overall activity impairment, %	26.23 (26.42)	18.52 (22.53)	30.13 (27.42)	< 0.001

*QoL* quality of life, *PCS* Physical component summary, *MCS* Mental Component Summary, *EQ-5D* EuroQol 5 Dimension. Continues variables were expressed as mean (SD). T-test was used for continuous variables

Table 5 shows the adjusted means examining the effect of having sleep symptoms on QoL scores after controlling for clinical characteristics. The adjusted PCS, MCS, EQ-5D of the participants with any sleep disorders were lower than those of the participants without and adjusted overall activity impairment of participants with sleep disorders was significantly higher than that of participants without. This result was the same for the symptoms of waking up to go to the bathroom and daytime sleepiness. On the other hand, there was no difference in the adjusted means PCS, MCS, and overall activity impairment between participants with and without the symptom of waking up too early.

**Table 5:** Adjusted means examining the effect of having sleep symptoms on QoL scores after controlling for clinical characteristics

Sleep symptoms	Health outcomes	Adjusted mean (95% CI)	Adjusted mean (95% CI)	*p*-value
Any sleep symptoms		No (n = 115)	Yes (n = 227)	
	PCS	50.14 (48.86–51.41)	47.99 (47.10–48.88)	0.009
	MCS	51.32 (49.56–53.08)	46.54 (45.31–47.76)	< 0.001
	EQ-5D	0.84 (0.81–0.87)	0.77 (0.75–0.79)	< 0.001
	Overall activity impairment, %	18.87 (15.16–23.48)	27.26 (23.46–31.68)	0.009
Waking up to go to the bathroom		No (*n* = 221)	Yes (*n* = 121)	
	PCS	49.67 (48.78–50.57)	46.96 (45.73–48.19)	< 0.001
	MCS	49.29 (48.03–50.54)	46.06 (44.33–47.79)	0.004
	EQ-5D	0.81 (0.79–0.84)	0.76 (0.73–0.78)	0.002
	Overall activity impairment, %	20.73 (17.81–24.14)	31.24 (25.35–38.5)	0.003
Daytime sleepiness		No (*n* = 247)	Yes (*n* = 95)	
	PCS	49.21 (48.36–50.06)	47.41 (45.99–48.83)	0.038
	MCS	49.51 (48.34–50.68)	44.6 (42.64–46.55)	< 0.001
	EQ-5D	0.82 (0.80–0.84)	0.73 (0.70–0.77)	< 0.001
	Overall activity impairment, %	22.30 (19.39–25.64)	29.92 (23.73–37.71)	0.038
Waking up too early (such as before the alarm clock)		No (*n* = 273)	Yes (*n* = 69)	
	PCS	48.94 (48.14–49.75)	47.8 (46.13–49.46)	0.233
	MCS	48.51 (47.39–49.64)	46.7 (44.37–49.03)	0.178
	EQ-5D	0.80 (0.79–0.82)	0.75 (0.71–0.79)	0.02
	Overall activity impairment, %	23.83 (20.88–27.19)	26.58 (20.19–34.99)	0.493

*QoL* quality of life, *PCS* Physical component summary, *MCS* Mental Component Summary, *EQ-5D* EuroQol 5 Dimension. The QoL scores of the presence or absence of specific sleep symptoms were compared by multivariable analysis adjusting for age, sex, marital status, level of education, charlson comorbidity index, smoking status, alcohol use, BMI category, taking steps to lose weight, duration of diabetes, HbA1c level, use of injectable medications and experience of hypoglycemia. We selected specific sleep symptoms of waking up to go to the bathroom, daytime sleepiness and waking up too early (such as before the alarm clock) for multivariable analysis, because prevalence rates of these symptoms were over 20%

## Discussion

This cross-sectional study showed the association between sleep disorders or specific sleep symptoms and QoL in patients with T2D. It revealed that 66.4% people with T2D suffer from sleep symptoms and that their QoL, including HRQoL and overall activity impairment, was worse than those of respondents without symptoms. This finding correlates with the results of previous studies [6–10, 27]. Also, this study revealed that waking up to go to the bathroom and daytime sleepiness are associated with worse QoL in people with T2D. Because to maintain and improve the QoL of people with diabetes is important goal, it should be recognized that the patients may have sleep disorders, especially the specific sleep symptoms discussed here.

Sleep disorders affect sleep quantity and quality. Sleep quantity is connected to the risk of lifestyle-related diseases, such as diabetes [28] and nonalcoholic fatty liver disease [29]. Also, sleep quantity affects QoL; a survey of more than 200,000 people in the United States with chronic illnesses has confirmed that the U-shape relationship between sleep time and the prevalence of decreased HRQoL, suggesting that decrease in sleep time leads to a decline in HRQoL [30]. This relationship has been observed similarly in other countries in people with various diseases [31–34]. Relatedly, sleep quality is also an important matter, and one of the problems of sleep quality is sleep fragmentation. Sleep fragmentation leads to metabolic disorders through activating autonomic sympathetic nerves [35]. The activation of autonomic sympathetic nerves leads to cardiovascular disease through changes in hemodynamics, vasoconstriction, and blood coagulation status [36]. Sleep fragmentation is also associated with psychological symptoms, such as depression [37]. Furthermore, the frequency of sleep disturbance is closely linked with the severity of self-reported symptoms in healthy individuals [38].

In this study, waking up to go to the bathroom is associated with poor QoL, including PCS, MCS, EQ-5D, and overall activity impairment, whereas waking up too early (such as before the alarm clock) is not associated with poor QoL, including PCS, MCS, and overall activity impairment. These results might be because of the difference in the type of sleep disorder. Because of the interruption of sleep, sleep fragmentation occurs when waking up to go to the bathroom. Waking up to go to the bathroom has close association with nocturia. Many people with T2DM are suffered from nocturia [39]. Many risk factors, including male sex, hypertension, high B-type natriuretic peptide level, low vegetable intake and early bedtime are reported [39, 40]. Dietary education [41] and medication intervention might be useful to reduce these risk factors. On the other hand, a decrease in sleep time occurs when waking up too early (such as before the alarm clock). Previous studies revealed that the effect of sleep fragmentation on mental and physical health is more severe than that of sleep quantity [42, 43].

This study also revealed that daytime sleepiness is associated with poor QoL. This correlates with studies that show that elderly people with hypertension and daytime sleepiness have a reduced QoL [44]. The decline of sleep quantity, which reduces the performance of higher cognitive processes, [45] might cause HRQoL to decline through daytime sleepiness.

This study has some limitations. Data from the NHWS are self-reported and did not include medical records or physician’s reports, and so no verification of diagnoses can be conducted. Moreover, the representativeness of each subsample is unknown. However, these data have been used in various publications, including data of T2D [46, 47]. Causal relationships between sleep symptoms and health outcomes cannot be assumed. The Japan NHWS 2016 had been completed when we started to analyze it. Therefore, the sample size was not predefined.

## Conclusion

In this study, about two-thirds of the people with T2D suffered from sleep disorders, and there is a relationship between sleep-related symptoms, especially waking up to go to the bathroom and daytime sleepiness, and HRQoL or labor productivity. It is, therefore, important to focus on sleep disorders, especially on symptoms of waking up to go to the bathroom or daytime sleepiness, in order to maintain and improve QoL of people with diabetes. Further studies focusing on sleep symptoms are needed for improvement QoL of people with diabetes.

## Data Availability

The data that support the findings of this study are available from Kantar Health but restrictions apply to the availability of these data, which were used under license for the current study, and so are not publicly available. Data are however available from corresponding authors, Michiaki Fukui, upon reasonable request and with permission of Kantar Health.

## Abbreviations

QoL: Quality of life
T2D: Type 2 diabetes
NHWS: National Health and Wellness Survey
HRQoL: Health-related QoL
PCS: Physical component summary
MCS: Mental component summary
EQ-D: EuroQol 5 Dimension
LSR: Lightspeed Research
BMI: Body mass index
CCI: Charlson comorbidity index
SF-36: 36-Item short-form
WPAI: Work Productivity and Activity Impairment
